# Supplementary material for: Complexome profiling of the Chlamydomonas psb28 mutant reveals TEF5 as an early PSII assembly factor
Source: Plant Cell. 2025 Mar 17;37(6):koaf055. doi: 10.1093/plcell/koaf055 (PMC12164590; doi:10.1093/plcell/koaf055)
Supplement: koaf055_Supplementary_Data [file koaf055_supplementary_data.zip › psb28_interactive.zip!/plots/intensity/qualityPlots/peptides/COP1_Cre01.g002500.t1.2.html]

### Cre01.g002500.t1.2

Trivial name: COP1  
SeqCoverage [median]: 63.00  
Mass: 26.58 kDa  
  
 

| Sequence | MissedCleavage | Mass [Da] | wtIntensity | muIntensity | Score |
| --- | --- | --- | --- | --- | --- |
| APTKPTGIVAGLHLDV | 1 | 1587.90 | 4718.44 | 58302.76 | 103.39 |
| GFEATYDVK | 0 | 1028.48 | 11565.90 | 15757.86 | 157.86 |
| GLVGTLTHK | 0 | 924.54 | 1428.91 | 8368.96 | 95.91 |
| IVAEVKPGK | 1 | 939.58 | 5813.89 | 8307.99 | 133.55 |
| IVAEVKPGKVAGLKPK | 3 | 1633.03 | 0.00 | 734.14 | 66.28 |
| LANPGAKPGIEVEYK | 1 | 1584.85 | 15223.47 | 28431.46 | 120.53 |
| LDQVIELNGGK | 0 | 1184.64 | 256.53 | 644.81 | 77.66 |
| LDQVIELNGGKEK | 1 | 1441.78 | 386.79 | 2030.85 | 134.24 |
| LKLEFDSK | 1 | 978.54 | 3177.86 | 7884.08 | 129.42 |
| LKVADNISVK | 1 | 1085.64 | 0.00 | 1230.58 | 104.43 |
| SISGEYDVSK | 0 | 1083.51 | 947.66 | 530.60 | 85.73 |
| SISGEYDVSKK | 1 | 1211.60 | 20102.74 | 47249.74 | 152.67 |
| SKDFSIEK | 1 | 952.49 | 764.62 | 1520.39 | 105.10 |
| SKDFSIEKK | 2 | 1080.58 | 2032.25 | 4830.05 | 143.40 |
| TELLPSPEVQWK | 0 | 1425.75 | 7436.64 | 15505.35 | 119.68 |
| VADNISVK | 0 | 844.47 | 21928.76 | 60335.27 | 160.63 |
| VGQSWAK | 0 | 774.40 | 4109.27 | 785.99 | 128.48 |
| YSQAAGPSLIHEVEPSK | 0 | 1811.91 | 2124.08 | 4754.99 | 96.99 |
| YSQAAGPSLIHEVEPSKK | 1 | 1940.00 | 10271.58 | 26015.08 | 105.89 |
|  |  |  | 112289.4 | 293221.0 |  |
